# Health-related quality of life and psychological distress among cancer survivors in Southeast Asia: results from a longitudinal study in eight low- and middle-income countries

**DOI:** 10.1186/s12916-016-0768-2

**Published:** 2017-01-13

**Authors:** The ACTION Study Group, M. L. Kimman, S. Jan, S. A. E. Peters, C. H. Yip, C. A. Ngelangel, N. Bhoo-Pathy, M. Woodward

**Affiliations:** 1 The George Institute for Global Health, University of Sydney, Level 10, King George V Building, 83-117 Missenden Rd, Camperdown, NSW 2050 Australia; 2 Department of Clinical Epidemiology and Medical Technology Assessment, Maastricht University Medical Centre, Minderbroedersberg 4-6, Maastricht, 6211 LK The Netherlands

**Keywords:** Health-related quality of life, Psychological distress, Cancer, Survivorship, Low- and middle-income countries, Southeast Asia, Observational study

## Abstract

**Background:**

A better understanding of health-related quality of life (HRQoL) and psychological distress in cancer survivors can raise awareness, promote the development of policies in cancer survivorship care, and facilitate better targeted use of limited resources in low- and middle-income countries (LMICs). The main objectives of this paper were therefore to assess HRQoL and the prevalence of psychological distress amongst cancer survivors in Southeast Asia and identify risk factors of these outcomes.

**Methods:**

The ACTION study was a longitudinal study in eight LMICs in Southeast Asia with 5249 first time cancer survivors followed up at 1 year after diagnosis. HRQoL was assessed using the EORTC QLQ-C30 and EQ-5D. Psychological distress (anxiety and depression) was assessed using the Hospital Anxiety and Depression Scale. General linear models and multiple logistic regression were used to identify independent predictors of HRQoL and psychological distress.

**Results:**

One year after diagnosis, the mean EORTC QLQ-C30 global health score for survivors was 66.2 out of 100 (SD 22.0), the mean index score on the EQ-5D was 0.74 (SD 0.23), 37% of survivors had at least mild levels of anxiety, and 46% showed at least mild levels of depression. Poorest HRQoL and highest prevalence of anxiety and depression were seen in patients with lung cancer and lymphomas, while highest scores and least psychological distress were seen in female patients with breast and cervical cancer. The most significant predictor of poor HRQoL and psychological distress outcomes was cancer stage at diagnosis. Age, co-morbidities, treatment, and several socioeconomic factors were associated with HRQoL and psychological distress.

**Conclusions:**

Cancer survivors in LMICs in Southeast Asia have impaired HRQoL and substantial proportions have psychological distress. Patients with advanced cancer stages at diagnosis and those in a poor socioeconomic position were most at risk of such poor outcomes. Supportive interventions for cancer patients that address wider aspects of patient wellbeing are needed, as well as policies that address financial and other barriers to timely treatment.

**Electronic supplementary material:**

The online version of this article (doi:10.1186/s12916-016-0768-2) contains supplementary material, which is available to authorized users.

## Background

Cancer is the world’s second leading cause of death and a major cause of disability. In 2013, an estimated 8.2 million individuals died of cancer, equating to 15% of all deaths globally [1]. While cancer survival rates are improving in high-income countries, cancer mortality rates are particularly high in low- and middle-income countries (LMICs), largely because of delays in diagnosis leading to presentation with advanced disease [2]. Breast cancer survival rates, for instance, range from 80% or higher in North America, Sweden and Japan to around 60% in middle-income countries and below 40% in low-income countries [3]. In addition, cancer affects populations in LMICs at relatively young ages, resulting in long periods of ill-health, great loss of productivity, and premature deaths [2, 4]. The burden of cancer continues to increase because of the ageing and growth of the population alongside increasing levels of cancer-causing behaviors such as smoking, physical inactivity, and unhealthy dietary habits [2]. Thus, cancer threatens health and economic development in LMICs and requires urgent attention [5–8].

The ACTION study was a longitudinal study of 9513 newly diagnosed cancer patients in eight LMICs in Southeast Asia, set up to prospectively assess the impact of cancer on households’ economic wellbeing and health [9]. Results of the study reported thus far have demonstrated that families living in Southeast Asia struggled to manage the costs associated with cancer care; over half the households faced catastrophic out-of-pocket payments (defined as spending more than 30% of household income for cancer-related costs) in the year after diagnosis [10, 11].

In addition to economic outcomes, key outcomes considered were health-related quality of life (HRQoL) and psychological distress 1 year after diagnosis [9]. While treatment has generally been completed a year after diagnosis, the cancer burden may still be significant due to short- and long-term sequelae that impair HRQoL [12, 13]. Cancer survivors are at increased risk of cancer-related fatigue and psychological symptoms [14–19]. Poor health is an important barrier to get back to work or take up activities of daily living after treatment [20, 21], thereby putting individuals and families at risk of impoverishment. However, few studies have been conducted in LMICs, and awareness of health issues affecting cancer survivors is still low [22, 23]. A better understanding of HRQoL and psychological distress in cancer survivors can raise awareness, promote the development of policies in cancer survivorship care, and facilitate better targeted use of limited resources [8].

The main objectives of this study were to assess HRQoL and the prevalence of psychological distress amongst cancer survivors in LMICs in Southeast Asia, 1 year after diagnosis. Secondary objectives were to identify demographic, clinical and socioeconomic predictors of poor HRQoL and psychological distress.

## Methods

### Setting and participants

The ACTION study was a prospective longitudinal study in which cancer patients from eight LMICs of ASEAN (Cambodia, Indonesia, Laos, Malaysia, Myanmar, Philippines, Thailand and Vietnam) were invited to participate. Detailed methods have been published previously [9]. In brief, 9513 first-time cancer patients were consecutively recruited from 47 sites, of which more than 95% were public hospitals. Participants, aged 18 years and over, were interviewed by study staff at baseline (within 12 weeks after clinical diagnosis) and at 3 and 12 months after diagnosis. One year after diagnosis, 29% (*n* = 1993) of the initial study population had died and 24% (*n* = 2271) were lost to follow-up. The remaining 5249 survivors were contactable at 12 months and were included in this report.

The ACTION study was approved by the University of Sydney Human Research Ethics Committee. Approvals from local institutional ethics committees and other regional or national regulatory bodies were obtained prior to the initiation of the study in any site (Additional file 1). Written informed consent, complying with local, regional, and national requirements, was obtained from all participants prior to entry into the study.

### Patient-reported health outcomes

Cancer-specific HRQoL was assessed by the European Organisation for Research and Treatment of Cancer (EORTC) Quality of Life Questionnaire (QLQ) C30 (version 3.0). Official translations were available for all countries except Laos and Cambodia [24]. The following were assessed: global health status, role function, cognitive function, physical function, emotional function, social function, fatigue, nausea and vomiting, pain, dyspnea, insomnia, appetite, constipation, and diarrhea. Scores are presented on a linear scale of 0 to 100. Higher scores correspond to better HRQoL in the function and global health scales, whereas higher scores in symptom scales and items represent more problems with symptoms [25].

Generic HRQoL was assessed using the EuroQol-5 dimensions questionnaire (EQ-5D) [26], which comprises five items relating to problems in mobility, self-care, usual activities, pain and discomfort, and anxiety and depression. The EQ-5D index provides a valuation of HRQoL in which full health is scored at 1, and death is 0 [27]. Official translations were provided by the EuroQoL group for all countries except Laos and Cambodia, covering 96% of the study population.

Psychological distress was measured using the Hospital Anxiety and Depression Scale (HADS), which measures generalized anxiety and depression experienced during the past week with two subscales: anxiety (HADS-A) and depression (HADS-D) [28]. The HADS is a self-report instrument and has been widely used in cancer patients [29]. A cut-off of ≥ 8 on the HADS-D scale and ≥ 9 on the HADS-A was used to indicate cases of least mild depression and anxiety, respectively [29]. Official translations were available for Indonesia, Malaysia, Philippines and Thailand, covering 63% of the study population.

In cases where a formal translation was not available, questionnaires were translated using forward-translation and back-translation following WHO guidelines for the process of translation of questionnaires [30].

### Predictor variables

We considered a range of demographic, socioeconomic and clinical variables as putative predictors of HRQoL and psychological distress. The demographic and socioeconomic variables considered were collected through self-reported questionnaires and relate to the time of entry to the study (baseline). Demographic variables considered were age, sex, marital status, and country of residence. Socioeconomic variables considered were household income (grouped into low (0–75% of mean national income), middle (75–125%) and high income (>125%)), economic hardship (whether in the 12 months previous to baseline they were unable to make necessary household payments or needed assistance to do so [31]), employment status (paid work), health insurance status, and highest level of education attained. Clinical variables obtained from medical records at baseline comprised cancer site, cancer stage at diagnosis (clinical TNM classification), and pre-existing chronic conditions. Treatment modality was ascertained at the end of study (12 months after baseline).

### Statistical analyses

Descriptive statistics were used to report the distribution of demographic, socioeconomic and clinical characteristics of the full study population, and separately by sex. The distribution of HRQoL scores and prevalence of anxiety and depression at 12 months was determined for the study population, the most common cancer types (those with more than 200 cases), and other potential predictors of these outcomes. Evidence-based guidelines for the interpretation of differences in EQ-5D and QLQ-C30 scores were used [32, 33]. For example, an observed mean difference of < 4 points on the QLQ-C30 global health scale was considered trivial and unlikely to have clinical relevance [34]. General linear models for HRQoL endpoints and multiple logistic regression for anxiety and depression were used to determine the association between predictor variables and study outcomes. To limit the number of tests and its associated increased likelihood of a type I error occurring, multivariable analyses were conducted for selected outcomes; global health, physical function, emotional function, fatigue, and pain. These outcomes are hypothesized to be most distinctive for the long-term health of different subgroups of cancer patients [21]. Stratified analyses explored the impact of predictor variables across the most common cancer types.

## Results

### Study sample

Characteristics of the 5249 survivors followed-up at 12 months are shown in Table 1; Additional file 2: Table S1A details the characteristics by sex. The mean age of the study population was 52 years (range, 18–100) and 69% were female. Almost half of the participants (*n* = 2365, 45%) were from lower middle-income countries (Laos, Indonesia, Vietnam, and the Philippines), followed by upper middle-income countries (Malaysia and Thailand; *n* = 2199, 42%) and low-income countries (Cambodia and Myanmar; *n* = 685, 13%).

**Table 1:** Demographic, socioeconomic, and clinical characteristics of the study population (*n* = 5249)

Characteristic	All	
	N	%
Age, years		
< 45	1569	30
45–54	1577	30
55–64	1369	26
≥ 65	732	14
Missing	2	<1
Sex		
Male	1618	31
Female	3631	69
Marital status		
Married	4047	77
Unmarried	1202	23
Level of education		
0–6 years (primary)	1932	37
7–12 years (secondary)	2169	41
> 12 years (tertiary)	1148	22
Country of residence		
Cambodia	131	3
Indonesia	673	13
Laos	56	1
Malaysia	1361	26
Myanmar	554	11
Philippines	458	9
Thailand	838	16
Vietnam	1178	22
Household income (of mean national income)		
Low	1643	31
Med	1048	20
High	1815	35
Do not know/missing	743	14
Health insurance status		
Yes	2249	43
None	2999	57
Missing	1	< 1
Experienced economic hardship in the year before diagnosis		
Yes	2643	50
No	2605	50
Missing	1	< 1
Paid work (patient level) before diagnosis (self-employed or for a wage)		
Yes	2481	47
No	2768	53
Cancer site		
Mouth and pharynx	571	11
Esophagus	49	< 1
Stomach	143	3
Colon and rectum	552	11
Liver	26	< 1
Pancreas	26	< 1
Trachea, bronchus and lung	226	4
Melanoma	18	< 1
Breast	1654	32
Cervix	598	11
Uterus	127	2
Ovary	123	2
Prostate	27	< 1
Bladder	20	< 1
Lymphomas and multiple myeloma	241	5
Leukemia	195	4
Other malignant neoplasms	617	12
Missing	36	< 1
Cancer (TNM) stage at diagnosis		
Stage I	437	8
Stage II	1190	23
Stage III	984	19
Stage IV	561	11
None (hematological cancers)	436	8
Missing	1641	31
Treatment^a^		
Surgery	2931	56
Radiotherapy	2438	46
Chemotherapy	3550	68
Hormonal therapy	496	9
Pre-existing chronic conditions (as reported in medical files)		
0	4032	77
1	839	16
≥ 2	352	7
Missing	26	< 1

^a^Categories are not mutually exclusive since most patients received a combination of treatments

The most common cancer types were breast (32%), cervix (11%), mouth and pharynx (11%), colon and rectum (11%), and lymphomas (5%). Among patients with available data on cancer stage (n = 3172), 14% presented with stage I, 38% with stage II, 31% with stage III, and 17% with stage IV cancers, whereas hematological cancers were diagnosed in 436 patients (8%). Fifty-six percent of participants had surgery as part of treatment, 68% had chemotherapy and 46% had radiotherapy (not mutually exclusive).

In females, cancer of the breast (45%) and cervix (17%) were most common. Among males, mouth (23%) and colorectal (19%) cancer were most common. Of female participants, 35% presented with stage I or II cancer compared to only 18% of males.

### Patient-reported health outcomes

A year after diagnosis, the mean global health score for survivors was 66.2 (SD 22.0) on the QLQ-C30 (Table 2). Scores on function scales ranged from 73.7 (SD 26.6) for social functioning, to 79.0 (SD 22.8) for physical function and 86.2 (SD 20.7) for cognitive functioning. In terms of symptoms, highest scores (i.e., more symptoms) were reported for fatigue (24.7), pain (21.4), and insomnia (21.2). The mean index score on the EQ-5D was 0.74 (SD 0.23). In terms of psychological distress, a year after diagnosis, anxiety was seen in 37% of survivors and depression in 46%.

**Table 2:** Health-related quality of life (HRQoL) and psychological distress 1 year after diagnosis

	All cancers (*n* = 5249)	
HRQoL		
Cancer-specific HRQoL (EORTC QLQ-C30)	Mean	SD
Global health	66.2	22.0
Physical function	79.0	22.8
Emotional function	76.2	24.8
Role function	74.8	28.8
Cognitive function	86.2	20.7
Social function	73.7	26.6
Fatigue	24.7	25.2
Nausea/vomiting	10.7	18.9
Pain	21.4	25.4
Dyspnea	13.9	22.8
Insomnia	21.2	27.5
Appetite loss	20.0	27.9
Constipation	11.3	21.4
Diarrhea	7.8	17.1
Generic HRQoL (EQ-5D)		
Index score	0.74	0.23
Psychological distress	N	%
HADS-A: Anxiety	1933	37
HADS-D: Depression	2394	46

*HADS* Hospital Anxiety and Depression Scale

**Fig. 1:**
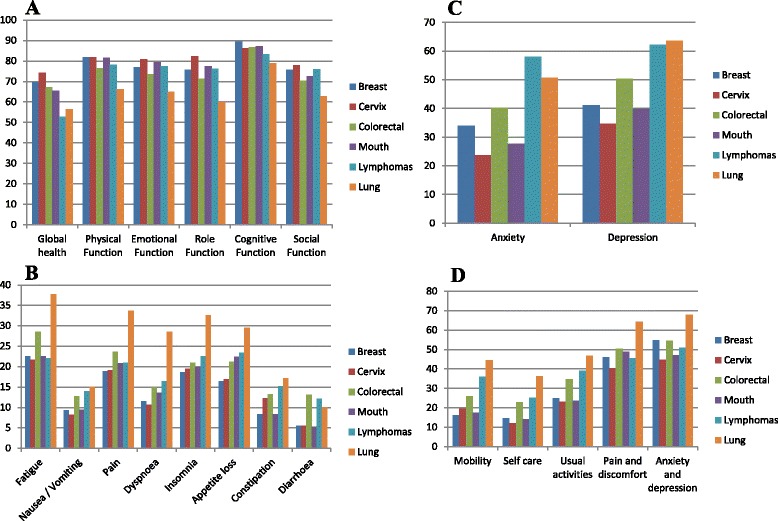
Health-related quality of life and psychological distress among cancer survivors (n = 5249) 1 year after diagnosis. **a** EORTC QLQ-C30 function scales, **b** EORTC QLQ-C30 symptom scales, **c** HADS anxiety and depression: proportion of survivors with anxiety and depression), and **d** EQ-5D domains: proportion of survivors with moderate or severe problems

Poorer outcomes, i.e., lowest HRQoL scores and highest symptom scores, were consistently seen in lung cancer and lymphoma patients (Fig. 1a–c, Additional file 2: Table S2A). Mean QLQ-C30 global health scores were respectively 56.5 (SD 23.2) and 52.8 (SD 27.2) for lung and lymphoma, compared to 74.2 (SD 17.7) and 69.7 (SD 19.4) for cancers of the cervix and breast. Similarly, the proportion of patients reporting moderate or severe problems on the EQ-5D domains was highest for lung cancer and lymphomas (Fig. 1d). The EQ-5D index scores for lung cancer and lymphomas were 0.63 (SD 0.24) and 0.69 (SD 0.24), respectively, compared to 0.76 (SD 0.21) and 0.78 (SD 0.21) for cancers of the breast and cervix, respectively. The proportion of patients with psychological distress was also highest among lung and lymphoma cancer patients; 60% of these patients report depression and anxiety was seen in 50% of lung cancer patients and 58% of lymphoma patients. Highest HRQoL scores, lowest symptom burden and lowest prevalence of anxiety and depression were seen in patients with cancer of the cervix.

HRQoL outcomes and psychological distress for subgroups of patients are presented in Table 3. Clinically meaningful differences in HRQoL scores (i.e., > 4 points difference on EORTC scales) were seen between participants older than 65 years compared to those younger than 45 years (e.g., EQ-5D index of 0.67 (SD 0.25) vs. 0.76 (SD 0.22), physical function of 72.6 (SD 28.0) vs. 81.9 (SD 21.5)). Older participants also reported more anxiety (45% vs. 36%) and depression (56% vs. 43%) than participants younger than 45 years. Male cancer survivors reported lower HRQoL scores on the EQ-5D, the QLQ-C30 function scales, and for fatigue and pain, but mean differences are considered trivial [34]. The prevalence of anxiety (42% vs. 35%) and depression (52% vs. 43%), however, is substantially higher in males than in females. The socioeconomic variables household income (low vs high), economic hardship, and not being in paid work were all associated with lower HRQoL scores, but differences were small. Nevertheless, a high income and paid work were associated with fewer cases of anxiety and depression. Health insurance was not associated with HRQoL and psychological distress.

**Table 3:** Demographic, socioeconomic, and clinical characteristics of the study population (*n* = 5249) and their associations with health-related quality of life (HRQoL) and psychological distress at 1 year after diagnosis as measured by the EORTC QLQ-C30, EQ-5D, and HADS

	HRQoL						Psychological distress	
	Generic HRQoL	Cancer-specific HRQoL (EORTC QLQ-C30)						
	EQ-5D index^a^	Physical function	Emotional function	Global health	Pain	Fatigue	Anxiety	Depression
	Mean (SD)	Mean (SD)	Mean (SD)	Mean (SD)	Mean (SD)	Mean (SD)	N (%)	N (%)
Age group, years								
< 45	0.76 (0.22)	81.9 (21.5)	77.8 (24.0)	67.0 (23.0)	19.2 (24.1)	21.6 (24.0)	570 (36)	678 (43)
45–54	0.75 (0.22)	80.9 (21.9)	76.7 (24.2)	67.5 (21.1)	20.7 (25.7)	23.5 (24.6)	513 (33)	663 (42)
55–64	0.73 (0.23)	77.9 (22.1)	75.8 (24.4)	65.9 (21.0)	22.1 (24.9)	26.1 (24.9)	519 (38)	644 (47)
≥ 65	0.67 (0.25)	70.5 (26.1)	72.6 (28.0)	62.6 (23.3)	31.4 (28.3)	31.4 (28.3)	330 (45)	408 (56)
Sex								
Female	0.75 (0.22)	80.1 (21.7)	76.8 (24.4)	66.2 (18.1)	20.2 (24.5)	23.9 (24.8)	1262 (35)	1560 (43)
Male	0.71 (0.24)	76.5 (24.8)	75.0 (25.5)	62.3 (20.0)	24.1 (27.0)	26.6 (26.0)	671 (42)	834 (52)
Marital status								
Married	0.74 (0.23)	79.2 (22.5)	76.5 (24.3)	66.7 (21.6)	21.5 (25.3)	24.6 (25.0)	1501 (37)	1845 (46)
Not married	0.73 (0.23)	78.3 (23.6)	75.5 (26.3)	64.9 (23.5)	21.1 (25.7)	24.9 (26.1)	432 (36)	549 (46)
Level of education								
0–6 years (primary)	0.73 (0.23)	77.8 (24.1)	77.1 (24.8)	68.1 (21.6)	21.5 (26.1)	24.2 (25.7)	641 (33)	849 (44)
7–12 years (secondary)	0.74 (0.23)	79.1 (21.8)	75.4 (24.2)	64.3 (21.4)	22.1 (24.8)	25.7 (24.7)	908 (42)	1088 (50)
> 12 years (tertiary)	0.75 (0.22)	80.8 (22.2)	76.3 (25.8)	66.8 (23.5)	19.9 (25.2)	23.7 (25.4)	384 (33)	457 (40)
Household income								
Low (0–75%)	0.71 (0.23)	76.5 (24.9)	74.0 (25.8)	64.9 (23.2)	23.3 (27.0)	25.4 (26.4)	712 (43)	818 (50)
Med (75–125%)	0.72 (0.23)	78.3 (21.8)	76.0 (24.3)	65.1 (22.1)	21.5 (24.9)	23.6 (24.2)	420 (40)	526 (50)
High (> 125%)	0.78 (0.22)	81.6 (21.2)	78.0 (24.4)	67.1 (20.7)	18.7 (23.8)	24.0 (24.5)	510 (28)	685 (38)
Health insurance								
Yes	0.75 (0.22)	78.6 (24.2)	76.9 (24.8)	68.0 (21.4)	22.7 (26.3)	25.9 (26.1)	813 (36)	1022 (46)
No	0.73 (0.23)	79.3 (21.6)	75.7 (24.8)	64.9 (22.4)	20.4 (24.6)	23.8 (24.5)	1120 (38)	1372 (46)
Economic hardship								
Yes	0.73 (0.23)	77.1 (24.9)	75.2 (25.9)	67.8 (22.0)	23.6 (27.5)	26.6 (27.1)	949 (36)	1217 (46)
No	0.75 (0.22)	80.9 (20.2)	77.3 (23.6)	64.7 (22.0)	19.2 (22.8)	22.8 (23.0)	984 (38)	1177 (45)
Paid work								
Yes	0.75 (0.22)	80.9 (22.2)	77.0 (24.9)	67.3 (21.8)	19.5 (25.1)	22.7 (24.8)	787 (32)	996 (40)
No	0.72 (0.23)	77.2 (23.2)	75.5 (24.7)	65.3 (22.2)	23.1 (25.6)	26.5 (25.5)	1146 (42)	1398 (51)
Cancer stage at diagnosis								
Stage I	0.81 (0.20)	85.7 (15.8)	82.3 (18.9)	74.2 (18.3)	12.5 (18.0)	16.6 (19.5)	99 (23)	141 (32)
Stage II	0.79 (0.21)	83.6 (18.6)	79.9 (22.8)	71.5 (18.9)	15.7 (21.1)	20.0 (22.0)	323 (27)	424 (36)
Stage III	0.75 (0.22)	79.6 (23.3)	76.0 (25.3)	69.0 (21.6)	20.4 (26.1)	24.0 (25.6)	296 (30)	400 (41)
Stage IV	0.65 (0.24)	69.8 (29.0)	63.4 (34.7)	57.0 (23.8)	32.6 (32.0)	36.6 (32.3)	265 (47)	327 (58)
None (hematological cancers)	0.67 (0.23)	77.4 (18.6)	77.9 (18.6)	44.7 (26.8)	21.1 (21.0)	22.1 (19.6)	308 (71)	313 (72)
Treatment^b^								
Surgery	0.78 (0.22)	82.1 (21.2)	78.7 (25.2)	69.9 (20.5)	17.9 (24.5)	22.1 (25.3)	1051 (36)	1051 (36)
No surgery	0.68 (0.23)	74.8 (24.1)	72.9 (24.1)	61.4 (23.2)	25.7 (25.8)	27.9 (24.9)	1123 (51)	1322 (60)
Radiotherapy	0.73 (0.22)	78.6 (22.5)	76.1 (24.4)	67.0 (21.7)	20.1 (24.9)	25.3 (25.0)	881 (36)	1123 (46)
No radiotherapy	0.74 (0.23)	79.2 (22.9)	76.2 (25.3)	65.5 (22.5)	22.5 (25.8)	24.0 (25.5)	1026 (38)	1248 (46)
Chemotherapy	0.73 (0.23)	78.6 (22.7)	75.2 (25.2)	65.0 (22.9)	21.7 (25.6)	25.3 (25.6)	1330 (38)	1652 (47)
No chemotherapy	0.76 (0.23)	79.7 (23.0)	78.4 (23.9)	69.0 (20.2)	20.2 (24.7)	23.1 (24.6)	580 (36)	732 (45)
Pre-existing chronic conditions								
0	0.74 (0.23)	79.6 (22.4)	76.8 (24.0)	66.3 (22.0)	21.1 (25.1)	24.0 (24.8)	1503 (37)	1853 (46)
1	0.74 (0.23)	79.0 (22.8)	77.2 (24.7)	66.5 (21.7)	20.4 (24.8)	24.2 (24.9)	266 (32)	357 (43)
≥ 2	0.67 (0.23)	71.5 (25.1)	67.5 (31.7)	64.8 (23.7)	27.5 (29.5)	34.6 (29.3)	159 (45)	177 (50)

^a^Index scores based on Thai tariff

^b^Categories are not mutually exclusive since most patients received a combination of treatments

Cancer stage at diagnosis and co-morbidities were associated with HRQoL and psychological distress. Directions were as expected; a more advanced cancer stage and more than two pre-existing chronic conditions were associated with lower HRQoL scores (all endpoints) and more cases of psychological distress.

Findings from the multivariable linear model showed that, in general, predictor variables, except treatment modalities (radiotherapy and chemotherapy), had a significant independent impact on HRQoL and psychological distress (Table 4). HRQol scores decreased, and the odds of psychological distress increased, with increasing age. Males had lower emotional function scores and higher odds of psychological distress compared to females. Other HRQoL outcomes were not affected by sex. Being married (vs. unmarried) positively impacted on physical and emotional function (QLQ-C30), but not on global health, the EQ-5D index, and psychological distress. Cancer stage at diagnosis was the strongest predictor with clinically meaningful reductions in HRQoL and psychological distress; stage IV and hematological cancers were predictive of severely impaired HRQoL (all endpoints) and the odds of having anxiety or depression. Having multiple chronic conditions was an important predictor for poor HRQoL and psychological distress. A high income (vs. low) and being in paid work positively impacted on most HRQoL outcomes and were associated with lower odds of psychological distress. Health insurance was associated with higher global health scores, but lower scores for physical function, pain and fatigue, and slightly higher odds of depression.

**Table 4:** Standardized betas of multiple linear regression analyses and logistic linear regression analyses evaluating the association of independent variables with HRQoL and psychological distress across all cancers (*n* = 5249)

Full model	EQ-5D index	Physical function	Emotional function	Global health	Pain	Fatigue	Anxiety	Depression
Age	−0.21 (−0.28 to −0.15)	−0.25 (−0.31 to −0.18)	−0.17 (−0.25 to −0.09)	−0.12 (−0.19 to −0.05)	0.20 (0.12 to 0.28)	0.27 (0.19 to 0.35)	1.01 (1.00 to 1.02)	1.01 (1.01 to 1.02)
Sex	−1.13 (−2.90 to 0.63)	−0.91 (−2.64 to 0.82)	−3.15 (−5.18 to −1.13)	0.73 (−1.01 to 2.47)	0.44 (−1.50 to 2.38)	1.16 (−0.79 to 3.11)	1.43 (1.24 to 1.62)	1.20 (1.02 to 1.37)
Marital status	0.65 (−1.17 to 2.48)	1.94 (0.16 to 3.73)	2.22 (0.13 to 4.31)	1.36 (−0.44 to 3.15)	−1.42 (−3.42 to 0.59)	−2.04 (−4.05 to −0.03)	1.18 (0.98 to 1.37)	0.93 (0.75 to 1.12)
Level of education								
Primary	Ref	Ref	Ref	Ref	Ref	Ref	Ref	Ref
Secondary	−2.70 (−4.55 to −0.86)	−2.17 (−3.98 to −0.37)	−5.16 (−7.27 to −3.04)	−5.63 (−7.45 to −3.81)	3.17 (1.14 to 5.20)	4.39 (2.36 to 6.43)	2.24 (2.04 to 2.44)	1.79 (1.60 to 1.97)
Tertiary	−4.07 (−6.30 to −1.84)	−2.82 (−5.00 to −0.63)	−6.04 (−8.60 to −3.48)	−3.68 (−5.88 to −1.48)	3.48 (1.02 to 5.94)	5.44 (2.97 to 7.90)	1.81 (1.56 to 2.05)	1.34 (1.12 to 1.57)
Household income								
Low	Ref	Ref	Ref	Ref	Ref	Ref	Ref	Ref
Medium	−0.83 (−2.90 to 1.24)	−0.20 (−2.23 to 1.83)	0.87 (−1.50 to 3.24)	−0.39 (−2.43 to 1.65)	0.22 (−2.06 to 2.50)	−0.27 (−2.56 to 2.01)	0.90 (0.68 to 1.11)	1.18 (0.98 to 1.39)
High	3.68 (1.78 to 5.58)	2.51 (0.64 to 4.37)	2.75 (0.57 to 4.94)	−0.09 (−1.97 to 1.79)	−2.49 (−4.59 to −0.39)	−0.39 (−2.49 to 1.71)	0.48 (0.28 to 0.69)	0.74 (0.55 to 0.93)
Health insurance	1.16 (−0.47 to 2.79)	−2.21 (−3.80 to −0.62)	0.09 (−1.78 to 1.96)	2.46 (0.85 to 4.06)	3.88 (2.09 to 5.67)	3.74 (1.94 to 5.54)	1.13 (0.95 to 1.30)	1.21 (1.05 to 1.38)
Economic hardship	−0.63 (−2.23 to 0.98)	−3.28 (−4.86 to −1.71)	−2.35 (−4.19 to −0.50)	2.44 (0.86 to 4.03)	3.41 (1.64 to 5.18)	3.85 (2.07 to 5.62)	0.81 (0.64 to 0.99)	0.94 (0.78 to 1.10)
Paid work	2.04 (0.40 to 3.68)	3.11 (1.50 to 4.72)	1.72 (−0.16 to 3.60)	2.76 (1.14 to 4.37)	−3.17 (−4.98 to −1.37)	−3.25 (−5.06 to −1.44)	0.66 (0.48 to 0.83)	0.68 (0.51 to 0.84)
Cancer stage at diagnosis								
Stage I	Ref	Ref	Ref	Ref	Ref	Ref		
Stage II	−1.43 (−4.06 to 1.20)	−2.09 (−4.66 to 0.48)	−1.37 (−4.38 to 1.65)	−2.14 (−4.73 to 0.45)	2.96 (0.07 to 5.86)	2.79 (−0.11 to 5.69)	1.30 (1.00 to 1.60)	1.12 (0.86 to 1.39)
Stage III	−3.42 (−6.18 to −0.66)	−4.45 (−7.15 to −1.75)	−4.77 (−7.94 to −1.61)	−4.09 (−6.81 to −1.37)	6.32 (3.29 to 9.36)	5.97 (2.93 to 9.02)	1.30 (0.99 to 1.61)	1.21 (0.93 to 1.49)
Stage IV	−13.34 (−16.40 to −10.27)	−14.73 (−17.73 to −11.73)	−18.61 (−22.13 to −15.10)	−15.14 (−18.16 to −12.11)	19.29 (15.91 to 22.67)	20.01 (16.63 to 23.40)	2.73 (2.40 to 3.07)	2.29 (1.98 to 2.60)
Hematological	−8.53 (−12.07 to −5.00)	−7.58 (−11.04 to −4.12)	−2.27 (−6.33 to 1.78)	−25.76 (−29.25 to −22.27)	7.89 (3.99 to 11.78)	5.68 (1.78 to 9.59)	5.05 (4.67 to 5.44)	3.06 (2.70 to 3.42)
Treatment								
Surgery	7.10 (5.34 to 8.85)	4.48 (2.77 to 6.20)	4.22 (2.21 to 6.23)	2.27 (0.54 to 4.00)	−4.81 (−6.74 to −2.88)	−3.29 (−5.22 to −1.35)	0.39 (0.21 to 0.57)	0.40 (0.23 to 0.58)
Radiotherapy	−0.44 (−2.01 to 1.14)	−0.27 (−1.81 to 1.27)	0.13 (−1.68 to 1.94)	0.10 (−1.45 to 1.66)	1.35 (−0.39 to 3.08)	0.53 (−1.21 to 2.27)	1.11 (0.94 to 1.28)	1.05 (0.89 to 1.20)
Chemotherapy	−3.47 (−5.29 to −1.65)	−0.58 (−2.36 to 1.20)	−1.56 (−3.65 to 0.52)	−1.20 (−3.00 to 0.59)	−0.45 (−2.45 to 1.56)	0.41 (−1.60 to 2.42)	0.93 (0.74 to 1.12)	1.02 (0.84 to 1.20)
No. of pre-existing chronic conditions	−2.32 (−3.44 to −1.19)	−2.51 (−3.61 to −1.41)	−2.92 (−4.21 to −1.63)	−0.22 (−1.33 to 0.89)	2.30 (1.06 to 3.54)	3.41 (2.17 to 4.65)	1.17 (1.06 to 1.29)	1.13 (1.02 to 1.24)

Age and number of pre-existing chronic conditions were entered as a continuous variable in the regression models. Other variables were sex: females vs. males; marital status: married vs. unmarried; level of education: primary vs. secondary vs. tertiary; household income: low vs. medium vs. high; health insurance: no vs. yes; economic hardship: yes vs. no; paid work: no vs. yes; cancer stage at diagnosis; stage I vs. stage II vs. stage III vs. stage IV; treatment: surgery vs. no surgery, radiotherapy vs. no radiotherapy; chemotherapy vs. no chemotherapy

Multivariable models stratified by cancer type showed that stage at diagnosis was the most important predictor of HRQoL and psychological distress across breast, cervical, lung, mouth, colorectal cancer, and lymphomas (Additional file 2: Tables S4A–F). In addition, a range of socioeconomic variables were associated with HRQoL endpoints and psychological distress across these cancer types. Results of the stratified analyses must be interpreted with caution because the models (except for breast cancer) were generally underpowered and could therefore only detect the strongest predictors.

## Discussion

One year after diagnosis, we identified impaired HRQoL and a prevalence of anxiety of 37% and depression of 46% amongst cancer survivors in LMICs in Southeast Asia. Differences in HRQoL and psychological distress were observed based on age, sex, household income, cancer type, and stage at diagnosis. In general, older patients, males, patients with lung cancer or lymphomas, an advanced stage at diagnosis, low income status, and those not in paid work reported lowest HRQoL scores and were most likely to report anxiety and depression. Female patients with cancer of the breast and cervix showed the most favorable outcomes reflecting a better prognosis for these type of cancers [2]. An advanced cancer stage at diagnosis was the strongest independent predictor of poor HRQoL outcomes and psychological distress. In addition, increasing age, having multiple chronic conditions, and several socioeconomic variables were identified as being independently associated with poor outcomes.

Inevitably, comparison of HRQoL and psychological distress with earlier studies is rough due to the varying settings, cancer types, and measures used. We are aware of few studies among cancer survivors in Southeast Asia or other LMICs [19, 35]. Most research on cancer survivorship has been conducted in high-income settings where patients were more likely to receive a timely diagnosis, optimal treatment, follow-up, and survivorship care. Survival rates, therefore, differ drastically between countries but, regardless of setting, many cancer survivors experience symptom burden, loss of HRQoL, and (at least mild) psychological distress in the first year after treatment [12, 13, 36, 37]. Cancer stage is widely recognized as an important clinical determinant of HRQoL in cancer survivors [12, 19, 37–42]. A diagnosis of lung cancer and having comorbid conditions are also associated with a high symptom burden and low HRQoL [12, 13, 36, 37]. In both low- and high-income settings, socioeconomic disparity in cancer survivors has been associated with poor HRQoL [12, 13, 35, 42–45] and psychological distress [46, 47]. Possible explanations may be poorer recognition of cancer symptoms and more barriers to access appropriate and timely healthcare services by patients with low socioeconomic status [48]. Patients with a poor socioeconomic position are also less likely to receive appropriate follow-up care and discuss concerns with their healthcare providers [49]. Our study has generally confirmed the relationship between poor socioeconomic position (e.g., a low household income, not being in paid work, and having experienced economic hardship) and impaired HRQoL and psychological distress, with one exception; primary education only was consistently associated with better study outcomes. While the absence of an association between education and HRQoL has been reported in certain cancer types and countries [36, 50], few studies find this inverse relationship between education and HRQoL [51]. To rule this out as a chance finding, this observation warrants further investigation. Another notable finding was that patients with health insurance were not necessarily better off than those without. These results, however, need to be interpreted with caution as we did not have data on benefit packages provided by the various health insurance schemes and associated processes of care. For example, Malaysia has achieved universal coverage that includes cancer care, but healthcare is provided through public health facilities that may have long waiting times [52].

Our study has a number of limitations. First, participation in the study was voluntary and, as such, the proportion of cases observed for individual cancers were not representations of population incidences. Clinicians responsible for enrolling patients into the study appear to have under-recruited those with the most virulent types of cancer such as lung and liver cancers. Second, by one year, 23% of the original study population had died and 24% were lost to follow-up. The high loss to follow-up is unfortunate, but not surprising, and may have introduced response bias. It is plausible that patients with poor HRQoL after treatment may have been less interested or too ill to continue participation; patients who completed the 12 months follow-up were more likely to have stage I or II cancer (Additional file 2: Table S1B). These first two limitations may have resulted in an underestimate of HRQoL and the prevalence of psychological distress in this patient population. Third, while HRQoL and psychological distress were measured upon study entry (within 12 weeks after clinical diagnosis), and at 3 and 12 months, we did not report change scores since the first two measurements are likely to reflect the type and timing of treatment (e.g., recovery from surgery, having second line treatment or just supportive care) as opposed to underlying HRQoL, and thus changes in scores may be transient fluctuations caused by treatment course. Fourth, while widely endorsed as one of the best available measures of anxiety and depression, the use of the HADS across languages and cultures has recently been criticized, e.g., there may be an unknown influence of linguistic and cultural factors on cut-points for anxiety and depression [53]. As such, there may be concerns that items in the translated version may differ conceptually to the original version. In our study, this risk may been pronounced in approximately 36% of the sample where official translations of the original questionnaires were not available and we had to undertake our own. Results presented here regarding prevalence must therefore be interpreted with caution. Nevertheless, the HADS was used for the purpose of subgroup comparisons and correlations rather than a screening tool, and therefore results provide useful insight into groups of patients with higher prevalence of anxiety and depression as well as risk factors. Finally, it was not possible to compare mean HRQoL scores of the cancer population to scores of the general population due to the lack of reference scores for Southeast Asia.

Despite limitations, our study is unique in being observational, including a large population of cancer survivors in an LMIC setting, and using a range of well-validated measures that characterize wellbeing in cancer patients. Until now, high-quality data on the quality of life lived with cancer have been lacking in ASEAN populations. Results from this study should encourage governments in the region to take action to develop national cancer control strategies and extend national health insurance initiatives to remove barriers to early diagnosis and prompt treatment [2, 8]. Awareness education and screening for detectable cancers may result in a larger proportion of patients presenting with early stage cancers that are more likely to be able to receive curative and less invasive treatment options, leading to cost savings and a reduction in mortality [54, 55], as well as to better HRQoL and lower levels of psychological distress among survivors, as found herein. Following (early) diagnosis, governments should ensure access to appropriate and quality treatment, including medication for cancer [6]. The focus of this paper, however, is on the needs of cancer survivors. Supportive and survivorship care, i.e., the prevention and management of the adverse effects of cancer and its treatment are increasingly recognized as critical components of quality cancer care and cancer control programs. Yet, in resource-constrained health systems, survivorship care is often overlooked [56–58]. Consensus statements and recommendations have been developed, mainly for breast cancer, to illustrate how health systems in LMICs can provide appropriate cancer care, including survivorship care, taking resource constraints into account [22, 56, 59]. Most recommendations can be generalized to other cancers. First, at the health system level, appropriate follow-up care (in terms of frequency and intensity) after completion of initial treatment is needed to monitor for possible recurrences or new cancers and adverse effects of treatments [22]. Scheduled follow-up visits, and the name of the health professional responsible for post-treatment care, should be documented in a patient’s individual care and survivorship plan [60]. This document should also include details of the patient’s treatment protocol so that potential specific treatment-related complications and long-term physical side effects can be considered [56, 59]. Importantly, assessment of psychosocial needs, including depression, anxiety, emotional distress, and changes to social roles, should be part of the follow-up care provided. Health professionals need (additional) education about the recognition and management of long-term physical complications and psychosocial complications of survivorship [22]. In addition, patients must be educated on symptoms of disease recurrence and lifestyle modifications to reduce cancer risk and improve quality of life [22, 59]. Our findings emphasize that patients in poor socioeconomic positions (e.g., low income, unemployed) and patients with late-stage cancers are at highest risk for poor HRQoL and psychological distress and therefore policies are needed to address the financial burden of treatment, including the expansion of national insurance programs and other social safety nets to offset the indirect costs incurred by patients and their families. Survivorship interventions should address wide aspects of wellbeing and are ideally implemented as part of routinely offered programs of care. Governments and research organizations should encourage this type of cancer (survivorship) research and accelerate the translation of research findings into clinical and public health practice [6].

## Conclusions

This study provides valuable insight into the cancer burden in terms of HRQoL and psychological distress and risk factors for poor outcomes in low- and middle-income settings. Improving cancer awareness, early detection, prompt treatment, and appropriate survivorship care are the major public health and clinical approaches to improve the health and wellbeing of cancer survivors. Importantly, this study demonstrates that supportive interventions for cancer patients that address wider aspects of patient wellbeing are needed, including government initiatives to address the economic burden associated with treatment. Results can be used to support policies geared towards survivorship care and inform research evaluating supportive interventions.

## Additional files

Additional file 1: Overview approvals local ethics boards ACTION Study. (DOCX 15 kb)
Additional file 2:
**Table S1A**. Demographic, socioeconomic and clinical characteristics of the study population by sex (*n* = 5249). **Table S1B.** Baseline demographic, socioeconomic, and clinical characteristics of the study population (*n* = 5249) and non-responders at 12 months (*n* = 2271). **Table S2A.** Health-related quality of life (HRQoL) and psychological distress 1 year after diagnosis. Presented for the most common cancer sites (more than 200 cases). **Table S4A.** Standardized betas of multiple linear regression analyses and logistic linear regression analyses evaluating the association of independent variables with HRQoL and psychological distress, for breast cancer patients (*N* = 1654). **Table S4B.** Standardized betas of multiple linear regression analyses and logistic linear regression analyses evaluating the association of independent variables with HRQoL and psychological distress, for cervix cancer patients (*n* = 598). **Table S4C.** Standardized betas of multiple linear regression analyses and logistic linear regression analyses evaluating the association of independent variables with HRQoL and psychological distress, for mouth and pharynx cancer patients (*n* = 571). **Table S4D.** Standardized betas of multiple linear regression analyses and logistic linear regression analyses evaluating the association of independent variables with HRQoL and psychological distress, for colorectal cancer patients (*n* = 552). **Table S4E.** Standardized betas of multiple linear regression analyses and logistic linear regression analyses evaluating the association of independent variables with HRQoL and psychological distress, for lung cancer patients (*n* = 226). **Table S4F.** Standardized betas of multiple linear regression analyses and logistic linear regression analyses evaluating the association of independent variables with HRQoL and psychological distress, for lymphoma patients (*n* = 241). (DOCX 91 kb)

## Abbreviations

EORTC QLQ-C30: European Organisation for Research and Treatment of Cancer Quality of Life Questionnaire C30
EQ-5D: EuroQol-5 dimensions
HADS: Hospital Anxiety and Depression Scale
HRQoL: health-related quality of life (HRQoL)
LMICs: low- and middle-income countries
