# Supplementary material for: Health-related quality of life and psychological distress among cancer survivors in Southeast Asia: results from a longitudinal study in eight low- and middle-income countries
Source: BMC Med. 2017 Jan 13;15:10. doi: 10.1186/s12916-016-0768-2 (PMC5234136; doi:10.1186/s12916-016-0768-2)
Supplement: Additional file 2: — Table S1A. Demographic, socioeconomic and clinical characteristics of the study population by sex (n = 5249). Table S1B. Baseline demographic, socioeconomic, and clinical characteristics of the study population (n = 5249) and non-responders at 12 months (n = 2271). Table S2A. Health-related quality of life (HRQoL) and psychological distress 1 year after diagnosis. Presented for the most common cancer sites (more than 200 cases). Table S4A. Standardized betas of multiple linear regression analyses and logistic linear regression analyses evaluating the association of independent variables with HRQoL and psychological distress, for breast cancer patients (N = 1654). Table S4B. Standardized betas of multiple linear regression analyses and logistic linear regression analyses evaluating the association of independent variables with HRQoL and psychological distress, for cervix cancer patients (n = 598). Table S4C. Standardized betas of multiple linear regression analyses and logistic linear regression analyses evaluating the association of independent variables with HRQoL and psychological distress, for mouth and pharynx cancer patients (n = 571). Table S4D. Standardized betas of multiple linear regression analyses and logistic linear regression analyses evaluating the association of independent variables with HRQoL and psychological distress, for colorectal cancer patients (n = 552). Table S4E. Standardized betas of multiple linear regression analyses and logistic linear regression analyses evaluating the association of independent variables with HRQoL and psychological distress, for lung cancer patients (n = 226). Table S4F. Standardized betas of multiple linear regression analyses and logistic linear regression analyses evaluating the association of independent variables with HRQoL and psychological distress, for lymphoma patients (n = 241). (DOCX 91 kb) [file 12916_2016_768_MOESM2_ESM.docx]

**Additional file 2.**

**Table S1A. Demographic, socioeconomic and clinical characteristics of the study population by sex (n=5249).**

| Characteristic | Females | | Males | |
| --- | --- | --- | --- | --- |
|  | N | % | N | % |
| Age, years |  |  |  |  |
| < 45 | 1118 | 31 | 451 | 28 |
| 45 – 54 | 1180 | 33 | 397 | 25 |
| 55 – 64 | 905 | 25 | 464 | 29 |
| ≥ 65 | 427 | 12 | 305 | 19 |
| Missing | 1 | <1 | 1 | <1 |
| Marital status |  |  |  |  |
| Married | 2692 | 74 | 1355 | 84 |
| Unmarried | 939 | 26 | 263 | 16 |
| Level of education |  |  |  |  |
| 0 – 6 years (primary) | 1424 | 39 | 508 | 31 |
| 7 – 12 years (secondary) | 1412 | 39 | 757 | 47 |
| > 12 years (tertiary) | 795 | 22 | 353 | 22 |
| Country of residence |  |  |  |  |
| Cambodia | 103 | 3 | 28 | 2 |
| Indonesia | 532 | 15 | 141 | 9 |
| Laos | 45 | 1 | 11 | <1 |
| Malaysia | 871 | 24 | 490 | 30 |
| Myanmar | 362 | 10 | 192 | 12 |
| Philippines | 294 | 8 | 164 | 10 |
| Thailand | 588 | 16 | 250 | 16 |
| Vietnam | 836 | 23 | 342 | 21 |
| Household income (of mean national income) |  |  |  |  |
| Low | 1111 | 31 | 532 | 33 |
| Med | 702 | 19 | 346 | 21 |
| High | 1264 | 35 | 551 | 34 |
| Don’t know/missing | 554 | 15 | 189 | 12 |
| Health insurance status |  |  |  |  |
| Yes | 2072 | 57 | 927 | 57 |
| None | 1558 | 43 | 691 | 43 |
| Missing | 1 | <1 |  |  |
| Experienced economic hardship in the year before diagnosis |  |  |  |  |
| Yes | 1770 | 49 | 835 | 52 |
| No | 1861 | 51 | 782 | 48 |
| Missing |  |  | 1 | <1 |
| Paid work (patient level) before diagnosis (self-employed or for a wage) |  |  |  |  |
| Yes | 1567 | 43 | 914 | 57 |
| No | 2064 | 57 | 704 | 43 |
| Cancer site |  |  |  |  |
| Mouth and pharynx | 196 | 5 | 375 | 23 |
| Oesophagus | 13 | <1 | 36 | 2 |
| Stomach | 53 | 2 | 90 | 6 |
| Colon and rectum | 244 | 7 | 308 | 19 |
| Liver | 9 | <1 | 17 | 1 |
| Pancreas | 10 | <1 | 16 | 1 |
| Trachea, bronchus and lung | 80 | 2 | 146 | 9 |
| Melanoma | 8 | <1 | 10 | <1 |
| Breast | 1645 | 46 | 9 | <1 |
| Cervix | 598 | 17 | - | - |
| Uterus | 127 | 4 | - | - |
| Ovary | 123 | 3 | - | - |
| Prostate | - | - | 27 | 2 |
| Bladder | 7 | <1 | 13 | <1 |
| Lymphomas and multiple myeloma | 99 | 3 | 142 | 9 |
| Leukaemia | 81 | 2 | 114 | 7 |
| Other malignant neoplasms | 319 | 9 | 298 | 18 |
| Missing | 19 | <1 | 17 | 1 |
| Cancer (TNM) stage at diagnosis |  |  |  |  |
| Stage I | 338 | 9 | 99 | 6 |
| Stage II | 993 | 27 | 197 | 12 |
| Stage III | 685 | 19 | 299 | 19 |
| Stage IV | 263 | 7 | 298 | 18 |
| None (haematological cancers) | 180 | 5 | 256 | 16 |
| Missing | 1172 | 32 | 469 | 29 |
| Treatment^1^ |  |  |  |  |
| Surgery | 2239 | 62 | 692 | 43 |
| Radiotherapy | 1700 | 47 | 738 | 46 |
| Chemotherapy | 2484 | 68 | 1066 | 66 |
| Hormonal therapy | 469 | 13 | 27 | 2 |
| Pre-existing chronic conditions (as reported in medical files) |  |  |  |  |
| 0 | 2751 | 76 | 1281 | 79 |
| 1 | 600 | 17 | 239 | 15 |
| ≥ 2 | 260 | 7 | 92 | 6 |
| Missing | 20 | <1 | 6 | <1 |

^1^Categories are not mutually exclusive since most patients received a combination of treatments.

**Table S1B. Baseline demographic, socioeconomic and clinical characteristics of the study population (n=5249) and non-responders at 12 months (n=2271).**

| Characteristic | Study population | | Non-responders | |
| --- | --- | --- | --- | --- |
|  | N | % | N | % |
| Age, years |  |  |  |  |
| < 45 | 1569 | 30 | 705 | 31 |
| 45 – 54 | 1577 | 30 | 704 | 31 |
| 55 – 64 | 1369 | 26 | 547 | 24 |
| ≥ 65 | 732 | 14 | 315 | 14 |
| Missing | 2 | <1 |  |  |
| Sex |  |  |  |  |
| Male | 1618 | 31 | 804 | 35 |
| Female | 3631 | 69 | 1467 | 65 |
| Marital status |  |  |  |  |
| Married | 4047 | 77 | 1765 | 78 |
| Unmarried | 1202 | 23 | 499 | 22 |
| Level of education |  |  |  |  |
| 0 – 6 years (primary) | 1932 | 37 | 881 | 39 |
| 7 – 12 years (secondary) | 2169 | 41 | 878 | 39 |
| > 12 years (tertiary) | 1148 | 22 | 503 | 22 |
| Country of residence |  |  |  |  |
| Cambodia | 131 | 3 | 21 | 1 |
| Indonesia | 673 | 13 | 1257 | 55 |
| Laos | 56 | 1 | 143 | 6 |
| Malaysia | 1361 | 26 | 179 | 8 |
| Myanmar | 554 | 11 | 211 | 9 |
| Philippines | 458 | 9 | 92 | 4 |
| Thailand | 838 | 16 | 368 | 16 |
| Vietnam | 1178 | 22 | 21 | 1 |
| Household income (of mean national income) |  |  |  |  |
| Low | 1643 | 31 | 939 | 41 |
| Med | 1048 | 20 | 393 | 17 |
| High | 1815 | 35 | 602 | 27 |
| Don’t know/missing | 743 | 14 | 337 | 15 |
| Health insurance status |  |  |  |  |
| Yes | 2249 | 43 |  | 54 |
| None | 2999 | 57 |  | 46 |
| Missing | 1 | <1 |  |  |
| Experienced economic hardship in the year before diagnosis |  |  |  |  |
| Yes | 2643 | 50 | 1367 | 60 |
| No | 2605 | 50 | 888 | 39 |
| Missing | 1 | <1 | 16 | 1 |
| Paid work (patient level) before diagnosis (self-employed or for a wage) |  |  |  |  |
| Yes | 2481 | 47 | 1116 | 49 |
| No | 2768 | 53 | 1149 | 51 |
| Cancer site |  |  |  |  |
| Mouth and pharynx | 571 | 11 | 221 | 10 |
| Oesophagus | 49 | <1 | 22 | 1 |
| Stomach | 143 | 3 | 58 | 3 |
| Colon and rectum | 552 | 11 | 172 | 8 |
| Liver | 26 | <1 | 23 | 1 |
| Pancreas | 26 | <1 | 8 | <1 |
| Trachea, bronchus and lung | 226 | 4 | 134 | 6 |
| Melanoma | 18 | <1 | 13 | <1 |
| Breast | 1654 | 32 | 575 | 25 |
| Cervix | 598 | 11 | 242 | 11 |
| Uterus | 127 | 2 | 33 | 2 |
| Ovary | 123 | 2 | 79 | 4 |
| Prostate | 27 | <1 | 16 | <1 |
| Bladder | 20 | <1 | 15 | <1 |
| Lymphomas and multiple myeloma | 241 | 5 | 97 | 4 |
| Leukaemia | 195 | 4 | 71 | 3 |
| Other malignant neoplasms | 617 | 12 | 390 | 17 |
| Missing | 36 | <1 | 102 | 5 |
| Cancer (TNM) stage at diagnosis |  |  |  |  |
| Stage I | 437 | 8 | 79 | 4 |
| Stage II | 1190 | 23 | 252 | 14 |
| Stage III | 984 | 19 | 361 | 19 |
| Stage IV | 561 | 11 | 254 | 14 |
| None (haematological cancers) | 436 | 8 | 168 | 9 |
| Missing | 1641 | 31 | 403 | 18 |
| Treatment^1,2^ |  |  |  |  |
| Surgery | 2931 | 56 | 898 | 41 |
| Radiotherapy | 2438 | 46 | 726 | 33 |
| Chemotherapy | 3550 | 68 | 1500 | 66 |
| Hormonal therapy | 496 | 9 | 75 | 3 |
| Pre-existing chronic conditions (as reported in medical files) |  |  |  |  |
| 0 | 4032 | 77 | 1750 | 77 |
| 1 | 839 | 16 | 351 | 16 |
| ≥ 2 | 352 | 7 | 86 | 4 |
| Missing | 26 | <1 | 84 | 4 |

^1^Categories are not mutually exclusive since most patients received a combination of treatments.

^2^ For non-responders the treatment variable refers to treatment modalities planned at baseline.

**Table S2A. Health-related quality of life and psychological distress one year after diagnosis. Presented for the most common cancer sites (more than 200 cases).**

|  | Breast  (n=1654) | | Cervix  (n=598) | | Mouth  (n=571) | | Colorectal  (n=552) | | Lung  (n=226) | | Lymphomas  (n=241) | |
| --- | --- | --- | --- | --- | --- | --- | --- | --- | --- | --- | --- | --- |
| **Health-related quality of life** |  |  |  |  |  |  |  |  |  |  |  |  |
| *Cancer-specific HRQoL (EORTC QLQ-C30)* | Mean | SD | Mean | SD | Mean | SD | Mean | SD | Mean | SD | Mean | SD |
| Global health | 69.7 | 19.4 | 74.2 | 17.7 | 65.6 | 19.3 | 67.1 | 21.3 | 56.5 | 23.2 | 52.8 | 27.2 |
| Physical function | 81.7 | 19.6 | 81.8 | 20.4 | 81.7 | 23.7 | 76.4 | 24.3 | 66.2 | 28.5 | 78.2 | 21.1 |
| Emotional function | 77.0 | 23.4 | 80.9 | 20.3 | 79.3 | 25.1 | 73.5 | 26.6 | 65.0 | 32.2 | 77.3 | 21.2 |
| Role function | 75.7 | 27.0 | 82.3 | 25.0 | 77.3 | 29.4 | 71.3 | 30.4 | 60.2 | 33.5 | 76.3 | 24.5 |
| Cognitive function | 89.5 | 17.5 | 86.3 | 18.6 | 87.3 | 22.0 | 86.7 | 21.3 | 78.8 | 27.9 | 83.3 | 19.3 |
| Social function | 75.8 | 25.1 | 78.0 | 25.2 | 72.6 | 27.4 | 70.2 | 28.1 | 62.9 | 30.1 | 75.9 | 23.8 |
| Fatigue | 22.5 | 23.7 | 21.7 | 23.2 | 22.5 | 25.4 | 28.5 | 26.6 | 37.8 | 30.7 | 22.1 | 22.4 |
| Nausea/vomiting | 9.3 | 17.0 | 8.3 | 19.7 | 9.4 | 19.0 | 12.7 | 20.1 | 15.0 | 21.3 | 14.0 | 19.3 |
| Pain | 18.9 | 23.1 | 19.2 | 23.4 | 20.9 | 26.5 | 23.7 | 26.0 | 33.7 | 31.2 | 21.0 | 22.5 |
| Dyspnoea | 11.5 | 20.5 | 10.7 | 18.9 | 13.6 | 24.4 | 14.9 | 22.8 | 28.6 | 30.3 | 16.5 | 23.2 |
| Insomnia | 18.7 | 26.2 | 19.5 | 25.5 | 19.8 | 28.1 | 21.0 | 27.8 | 32.6 | 31.2 | 22.5 | 25.3 |
| Appetite loss | 16.5 | 24.7 | 16.9 | 26.0 | 22.4 | 29.8 | 21.3 | 30.0 | 29.5 | 32.6 | 23.4 | 28.3 |
| Constipation | 8.4 | 18.2 | 12.3 | 23.6 | 8.3 | 19.8 | 13.2 | 22.7 | 17.1 | 26.0 | 15.2 | 22.3 |
| Diarrhoea | 5.6 | 14.1 | 5.5 | 15.0 | 5.3 | 14.9 | 13.2 | 21.2 | 10.0 | 21.0 | 12.2 | 19.5 |
|  |  |  |  |  |  |  |  |  |  |  |  |  |
| *Generic HRQoL (EQ-5D)* |  |  |  |  |  |  |  |  |  |  |  |  |
| Index score | 0.76 | 0.21 | 0.78 | 0.21 | 0.77 | 0.22 | 0.72 | 0.24 | 0.63 | 0.24 | 0.69 | 0.24 |
|  |  |  |  |  |  |  |  |  |  |  |  |  |
| **Psychological distress** | N | % | N | % | N | % | N | % | N | % | N | % |
| HADS-A: Anxiety | 563 | 34 | 207 | 35 | 158 | 28 | 221 | 40 | 114 | 50 | 150 | 62 |
| HADS-D: Depression | 679 | 41 | 141 | 24 | 228 | 40 | 277 | 50 | 143 | 63 | 140 | 58 |

**Table S4A.** **Standardised betas of multiple linear regression analyses and logistic linear regression analyses evaluating the association of independent variables with HRQoL and psychological distress, for breast cancer patients (N=1654).**

Age and number of pre-existing chronic conditions were entered as a continuous variable in the regression models. Other variables were sex: females vs males; marital status: married vs unmarried; level of education: primary vs secondary vs tertiary; household income: low vs medium vs high; health insurance: no vs yes; economic hardship: yes vs no; paid work: no vs yes; cancer stage at diagnosis; stage I vs stage II vs stage III vs stage IV; treatment: surgery vs no surgery, radiotherapy vs no radiotherapy; chemotherapy vs no chemotherapy.

**Table S4B.** **Standardised betas of multiple linear regression analyses and logistic linear regression analyses evaluating the association of independent variables with HRQoL and psychological distress, for cervix cancer patients (n=598).**

Age and number of pre-existing chronic conditions were entered as a continuous variable in the regression models. Other variables were sex: females vs males; marital status: married vs unmarried; level of education: primary vs secondary vs tertiary; household income: low vs medium vs high; health insurance: no vs yes; economic hardship: yes vs no; paid work: no vs yes; cancer stage at diagnosis; stage I vs stage II vs stage III vs stage IV; treatment: surgery vs no surgery, radiotherapy vs no radiotherapy; chemotherapy vs no chemotherapy.

**Table S4C.** **Standardised betas of multiple linear regression analyses and logistic linear regression analyses evaluating the association of independent variables with HRQoL and psychological distress, for mouth and pharynx cancer patients (n=571).**

Age and number of pre-existing chronic conditions were entered as a continuous variable in the regression models. Other variables were sex: females vs males; marital status: married vs unmarried; level of education: primary vs secondary vs tertiary; household income: low vs medium vs high; health insurance: no vs yes; economic hardship: yes vs no; paid work: no vs yes; cancer stage at diagnosis; stage I vs stage II vs stage III vs stage IV; treatment: surgery vs no surgery, radiotherapy vs no radiotherapy; chemotherapy vs no chemotherapy.

**Table S4D.** **Standardised betas of multiple linear regression analyses and logistic linear regression analyses evaluating the association of independent variables with HRQoL and psychological distress, for colorectal cancer patients (n=552).**

Age and number of pre-existing chronic conditions were entered as a continuous variable in the regression models. Other variables were sex: females vs males; marital status: married vs unmarried; level of education: primary vs secondary vs tertiary; household income: low vs medium vs high; health insurance: no vs yes; economic hardship: yes vs no; paid work: no vs yes; cancer stage at diagnosis; stage I vs stage II vs stage III vs stage IV; treatment: surgery vs no surgery, radiotherapy vs no radiotherapy; chemotherapy vs no chemotherapy.

**Table S4E.** **Standardised betas of multiple linear regression analyses and logistic linear regression analyses evaluating the association of independent variables with HRQoL and psychological distress, for lung cancer patients (n=226).**

Age and number of pre-existing chronic conditions were entered as a continuous variable in the regression models. Other variables were sex: females vs males; marital status: married vs unmarried; level of education: primary vs secondary vs tertiary; household income: low vs medium vs high; health insurance: no vs yes; economic hardship: yes vs no; paid work: no vs yes; cancer stage at diagnosis; stage I vs stage II vs stage III vs stage IV; treatment: surgery vs no surgery, radiotherapy vs no radiotherapy; chemotherapy vs no chemotherapy.

**Table S4F.** **Standardised betas of multiple linear regression analyses and logistic linear regression analyses evaluating the association of independent variables with HRQoL and psychological distress, for lymphoma patients (n=241).**

Age and number of pre-existing chronic conditions were entered as a continuous variable in the regression models. Other variables were sex: females vs males; marital status: married vs unmarried; level of education: primary vs secondary vs tertiary; household income: low vs medium vs high; health insurance: no vs yes; economic hardship: yes vs no; paid work: no vs yes; treatment: surgery vs no surgery, radiotherapy vs no radiotherapy; chemotherapy vs no chemotherapy.
